# Nested case-control study investigating factors affecting initial adoption of HIV self-testing among men who have sex with men in Guangzhou, China: amidst comprehensive service coverage

**DOI:** 10.3389/fpubh.2024.1483671

**Published:** 2025-01-07

**Authors:** Yuzhou Gu, JiaLi Yang, Yefei Luo, Lishan Zhan, Fanghua Liu, Wenting Zeng, Huifang Xu, Yongheng Lu, Yanshan Cai, Zhigang Han

**Affiliations:** ^1^Department of HIV/AIDS Control and Prevention, Guangzhou Center for Disease Control and Prevention, Guangzhou, China; ^2^School of Public Health, Guangzhou Medical University, Guangzhou, China; ^3^Huangpu District Center for Disease Control and Prevention, Guangzhou, China; ^4^Guangdong Association of STD & AIDS Prevention and Control, Guangzhou, China; ^5^Lingnan Partner Community Support Center, Guangzhou, China

**Keywords:** self-testing, HIV, men who have sex with men, Guangzhou, nested case-control

## Abstract

**Background:**

China has been exploring HIV self-testing (HIVST) among men who have sex with men (MSM) since 2014. Currently, both non-profit and commercial initiatives HIVST services have achieved comprehensive coverage. Investigating the factors influencing the initial adoption of HIVST among MSM in this context can help develop tailored HIVST service strategies of and further promote HIVST adoption among MSM communities.

**Methods:**

We selected 230 participants from a prospective cohort on HIV infection among MSM population in Guangzhou, China, who had no prior experience of HIVST. Among the study participants, 43 who initially adopted HIVST during the follow-up period were designated as the case group, while the rest comprised the control group. Electronic questionnaires were used for baseline and follow-up surveys to collect demographic information, sexual behavior characteristics and HIVST utilization in the past 6 months. Logistic regression models were applied to analyze the factors influencing the initial adoption of HIVST.

**Results:**

Among the study participants, 18.7% (43/230) initially adopted HIVST during the follow-up period. Multivariate logistic regression analysis revealed that individuals who primarily sought sexual partners through offline venues in the past 6 months (aOR = 5.28, 95% CI: 1.01–27.79), had more than one sexual partner in the past 6 months (aOR = 2.76, 95% CI: 1.33–5.74), engaged with more than two casual partners in the past 6 months (aOR = 3.02, 95% CI: 1.35–6.78), or had more than one regular partner (aOR = 3.37, 95% CI: 1.51–7.51) exhibited an increased likelihood of initiating HIVST.

**Conclusion:**

In the context of comprehensive promotion and coverage of HIVST service, the development of personalized, adaptable, and innovative HIVST strategies for MSM with a higher number of sexual partners, particularly those in regular partnerships and those seeking partners offline, may further increase the adoption of HIVST among MSM.

## 1 Introduction

Epidemiological surveillance data from the Chinese Center for Disease Control and Prevention indicate that, as of the end of 2022, China had approximately 1.22 million people living with HIV/AIDS (PLWHA). The majority of newly reported HIV infection cases stem from sexual transmission, wherein male-to-male sexual transmission constitutes 25.6% (1). Located in southern China, Guangzhou is a major city with a population of 14 million. In Guangzhou, male-to-male sexual transmission has emerged as the predominant pathway of HIV transmission, accounting for over half of the newly reported cases annually. Consequently, interventions aimed at men who have sex with men (MSM) are imperative due to their heightened susceptibility to HIV, rendering them among the most heavily impacted populations in China. Comprehensive interventions, encompassing mobilization of testing among MSM and promotion of treatment for PLWHA among MSM, play a pivotal role in curbing HIV transmission and stemming the epidemic’s dissemination. These endeavors hold paramount significance in managing the HIV epidemic, spanning beyond Guangzhou to the entirety of China (1).

HIV self-testing (HIVST) entails individuals independently collecting samples, conducting self-tests with HIV rapid test kits, and autonomously interpreting the results (2). Due to its convenient and private nature, HIVST has considerable potential for promoting testing in regions and demographics with insufficient offline HIV testing services (3–5). Previous studies suggest that HIVST enhances MSM populations’ inclination and frequency for HIV testing (6), diminishes risky behaviors (7), and heightens awareness of individual HIV infection status (8). Since 2014, the World Health Organization (WHO) has been fostering worldwide endeavors to investigate and advance HIVST within pivotal groups, including MSM (2). The WHO released guidelines on HIVST in 2016, specifically endorsing the incorporation of HIVST as a complementary strategy to conventional HIV testing services (9). By 2020, the utilization and implementation of HIVST had received official approval in a total of 57 countries and regions across the globe (10).

In 2014, China embarked on the exploration of HIVST. The Guangzhou Center for Disease Control and Prevention (Guangzhou CDC), along with the Lingnan Partners Community Support Center, a community-based organization (CBO), collaborated to offer an online platform for MSM individuals to apply for HIVST kits, submit their results, and receive interpretation and consultation (11–13). Consequently, this non-profit HIVST model was extensively promoted nationwide. Consequently, different CBOs in various regions effectively facilitated HIV testing among local MSM populations through this service model of HIVST (14, 15). Following its explicit incorporation in China’s “Thirteenth Five-Year Plan for HIV/AIDS Prevention and Control,” issued by the State Council Office in 2017 (16), which outlined the expansion of HIVST services through diverse channels like pharmacies and online test kit sales, the first urine-based HIVST kit secured regulatory approval and was introduced to the Chinese market in 2019 (17). In the same year, China published its inaugural “Guidebook for HIVST” (5), coinciding with the emergence of commercially-driven HIVST services spearheaded by test kit manufacturers and e-commerce platforms. Capitalizing on substantial advantages in supply channels and marketing, commercial HIVST services have progressively risen to prominence, comprising a substantial proportion of HIVST utilization in China. Consumption data indicates an approximate sale of 400 HIVST kits per hour on e-commerce platforms (18–20). A specific test kit manufacturer in China disclosed an annual sale of 1 million HIVST kits on a designated e-commerce platform (14).

Propelled by both non-profit and commercial initiatives, and facilitated through a range of online and offline channels, China’s HIVST services have transformed into a varied and accessible framework, characterized by wide coverage and significant adoption. Being among the pioneering adopters of HIVST in China, MSM individuals have attained remarkable levels of awareness, acceptance, and utilization (21, 22).

Studies have demonstrated that service accessibility significantly affects the utilization of HIVST (23). Nevertheless, despite the broad range of service provision channels and comprehensive coverage, some MSM individuals have not yet embraced the use of HIVST. This underscores the need, at the present juncture of widespread HIVST service availability, to elevate the utilization rate among MSM individuals. This requires a strategic transition from mere supply augmentation to the cultivation of tailored, inventive, and flexible service models designed for MSM with varying attributes (24–26). These models are intended to address the specific HIVST requirements of diverse subgroups within the MSM population.

Previous investigations into the determinants of HIVST utilization have predominantly consisted of cross-sectional studies, which possess a restricted capacity for causal inference. Many of these studies were conducted when HIVST coverage was relatively limited. The central emphasis was on strategies to achieve significant enhancements in HIVST rates (23, 27–29). Consequently, the majority of research findings provide minimal assistance for advancing promotion, especially after the marginal effects of HIVST service utilization have been attained.

Guangzhou, an early advocate of HIVST services for MSM in China, is additionally a bustling international trade center. At present, the provisioning and coverage of HIVST among the MSM population in Guangzhou have nearly reached a state of saturation. Therefore, this study employs a prospective cohort established within the MSM population in Guangzhou, adopting a nested case-control design. The study aims to investigate factors impacting the initial adoption of HIVST among MSM individuals. The objective is to produce scientific evidence guiding the development of tailored strategies aimed at further bolstering HIVST adoption among MSM communities. In turn, this endeavor fosters the cultivation of consistent testing habits and mitigates HIV transmission within the MSM population.

## 2 Methods

### 2.1 Study population and design

We employed a non-matched nested case-control design, the case and control groups were extracted from a prospective cohort on HIV infection among MSM population in Guangzhou.

This cohort was conducted between June 2019 to April 2022, utilizing HIV voluntary counseling and testing (VCT) clinics from 12 CDC and 2 CBO in Guangzhou as field sites. The recruitment process targeted HIV antibody-negative males, aged 18 or above, residing in Guangzhou, who had engaged in male-to-male sexual behavior within the past 6 months. Participants who voluntarily provided informed consent and completed the baseline questionnaire were included in the study. Every 3–6 months, participants were mobilized to return to VCT clinics for follow-up questionnaire surveys and HIV antibody tests. The follow-up ended for individuals who tested positive for HIV antibodies. This study obtained ethical approval from the Ethics Committee of Guangzhou CDC (Approval No: GZCDC-ECHR-2020P0044). All methods were performed in accordance with the relevant guidelines and regulations.

From this cohort, we selected all 230 participants who had no prior experience of HIVST during the baseline survey as the study subjects. Among them, 43 who initial adoption of HIVST during the follow-up period were designated as the case group. The control group consisted of 187 participants who going non-utilize of HIVST throughout the follow-up period.

### 2.2 Collection of demographic and behavioral information

Both baseline and follow-up data were gathered through electronic questionnaires that we designed ourselves. Trained interviewers conducted surveys with study participants, adhering to standardized protocols. The survey covered demographic information, sexual behavior characteristics from the previous 6 months, utilization of HIVST in the past 6 months, as well as pre-exposure prophylaxis (PrEP) and post-exposure prophylaxis (PEP) utilization within the same timeframe.

(1) Definition of behavioral characteristics variables for the case group: The questionnaire responses from the latest follow-up survey prior to the initial adoption of HIVST were employed to establish the variables related to behavioral characteristics for the case group. (2) Definition of behavioral characteristics variables for the control group: The questionnaire responses from the most recent follow-up survey were utilized to determine the variables related to behavioral characteristics for the control group participants.

### 2.3 Statistical analysis

Statistical analyses were performed using SPSS 26.0 software. Descriptive statistics included means and standard deviations for quantitative data in both the case and control groups, while categorical data were presented using frequencies and proportions. Non-matched nested case-control data analysis was performed using a non-conditioned logistic regression model. Preliminary univariate analysis was conducted to explore factors influencing the initial adoption of HIVST. The significant variables identified through univariate analysis were incorporated into separate multivariate models, with each predictor factor analyzed individually while controlling for potential confounders, to prevent multicollinearity from affecting our results. A significance level of *p* < 0.05 indicated statistical significance for differences.

### 2.4 Quality control

Prior to data collection, standardized training was provided to interviewers and follow-up personnel to ensure their familiarity with recruitment methods and procedures. Only those who successfully completed the training assessment were allowed to proceed. Baseline and follow-up surveys were administered using electronic questionnaires to prevent omissions, logical errors, and potential issues arising from alterations to paper-based questionnaires or data entry errors. Survey questionnaires were labeled with unique participant codes, and baseline and follow-up questionnaires were consistently archived. Designated personnel were responsible for verification and quality control of the questionnaires. The study did not involve patient names or identifiable information, and all data were treated with strict confidentiality measures.

## 3 Results

### 3.1 Demographic characteristics

A total of 230 MSM with no prior experience of HIVST were included in this study. The participants had an average age of 30.0 (SD 6.9) years, with the majority being unmarried (93.5%, 215/230) and employed (68.3%, 157/230). Among the study participants, 18.7% (43/230) initially adopted HIVST during the follow-up period and were classified as the case group; the remaining 81.3% (187/230) consistently did not use HIVST and were classified as the control group. Refer to Table 1 for details.

**Table 1:** Demographic characteristics.

Variable	Participants (*n* = 230)	Case group (*n* = 43)	Control group (*n* = 187)
Age, years (SD)	30.0 (6.9)	29.4 (6.7)	30.2 (6.9)
**Marital status**			
Not married	215 (93.5)	39 (90.7)	176 (94.1)
Married	15 (6.5)	4 (9.3)	11 (5.9)
**Occupation**			
Unemployed	31 (13.5)	6 (14.0)	25 (13.4)
Employed	157 (68.3)	29 (67.4)	128 (68.4)
Non-fixed occupation	42 (18.3)	8 (18.6)	34 (18.2)
**Personal monthly income (RMB)**			
0–5,000	75 (32.6)	16 (37.2)	59 (31.6)
5,001–10,000	74 (32.2)	14 (32.6)	60 (32.1)
>10,000	81 (35.2)	13 (30.2)	68 (36.4)
**Awareness of HIVST**			
No	0 (0)	0 (0)	0 (0)
Yes	230 (100)	43 (100)	187 (100)
**Primary method of seeking sexual partners in the last 6 months**			
Did not seek	53 (23.0)	3 (7.0)	50 (26.7)
Online platform	160 (69.6)	36 (83.7)	124 (66.3)
Offline venue	17 (7.4)	4 (9.3)	13 (7.0)
**Number of male sexual partners in the last 6 months**			
≤1	111 (48.3)	13 (30.2)	98 (52.4)
>1	119 (51.7)	30 (69.8)	89 (47.6)
**CAI with male sexual partners in the last 6 months**			
No	174 (75.7)	32 (74.4)	142 (75.9)
Yes	56 (24.3)	11 (25.6)	45 (24.1)
**Number of male casual partners in the last 6 months**			
≤2	191 (83.0)	30 (69.8)	161 (86.1)
>2	39 (17.0)	13 (30.2)	26 (13.9)
**CAI with male casual partners in the last 6 months**			
No	207 (90.0)	39 (90.7)	168 (89.8)
Yes	23 (10.0)	4 (9.3)	19 (10.2)
**Number of male regular partners in the last 6 months**			
≤1	188 (81.7)	29 (67.4)	159 (85.0)
>1	42 (18.3)	14 (32.6)	28 (15.0)
**CAI with male regular partners in the last 6 months**			
No	181 (78.7)	33 (76.7)	148 (79.1)
Yes	49 (21.3)	10 (23.3)	39 (20.9)
**Experienced sexual violence in the last 6 months**			
No or unsure	224 (97.4)	40 (93.0)	184 (98.4)
Yes	6 (2.6)	3 (7.0)	3 (1.6)
**Perpetrated sexual violence against others in the last 6 months**			
No or unsure	227 (98.7)	41 (95.3)	186 (99.5)
Yes	3 (1.3)	2 (4.7)	1 (0.5)
**PrEP used in the last 6 months**			
Yes	11 (4.8)	2 (4.7)	9 (4.8)
No	219 (95.2)	41 (95.3)	178 (95.2)
**PEP used in the last 6 months**			
Yes	8 (3.5)	3 (7.0)	5 (2.7)
No	222 (96.5)	40 (93.0)	182 (97.3)

### 3.2 Sexual behavioral characteristics

All 230 MSM participants demonstrated complete knowledge of HIVST. In the preceding 6 months, 24.3% (56/230) of participants engaged in male-to-male condomless anal intercourse (CAI). Furthermore, 10.0% (23/230) had CAI with male casual partners, and 21.3% (49/230) with male regular partners. Moreover, 51.7% (119/230) reported having more than one male sexual partner during this time. Among them, 17.0% (39/230) had over two male casual partners, and 18.3% (42/230) had more than one male regular partner within the same period.

In the previous 6 months, 2.6% (6/230) of participants reported experiencing sexual violence, and 1.3% (3/230) admitted perpetrating sexual violence against others during the same period. Utilization rates for PrEP and PEP in the past 6 months were 4.8% (11/230) and 3.5% (8/230), respectively.

### 3.3 Logistic regression analysis of factors influencing the initial adoption of HIVST

Univariate logistic regression analysis indicated that several factors were linked to the initial adoption of HIVST. These factors included seeking sexual partners through online platforms (OR = 4.84, 95%CI: 1.43–16.43) or offline venues (OR = 5.13, 95%CI: 1.02–25.82), having more than one male sexual partners in the past 6 months (OR = 2.54, 95% CI: 1.25–5.18), having more than two male casual partners in the past 6 months (OR = 2.68, 95% CI: 1.24–5.80), and having more than one male regular partner in the past 6 months (OR = 2.74, 95% CI: 1.29–5.83) (see Table 2).

**Table 2:** Logistic regression analysis of factors influencing the initial adoption of HIVST.

Variable	Initial adoption of HIVST (%)	Univariate analysis		Multivariate analysis*	
		*p*-value	OR (95%CI)	*p*-value	aOR (95%CI)
**Primary method of seeking sexual partners in the last 6 months**					
Did not seek	3 (5.7)	–	1.00	–	1.00
Online platform	36 (22.5)	0.01	4.84 (1.43 ~ 16.43)	0.01	4.85 (1.42 ~ 16.59)
Offline venue	4 (23.5)	0.05	5.13 (1.02 ~ 25.82)	0.05	5.28 (1.01 ~ 27.79)
**Number of male sexual partners in the last 6 months**					
≤1	13 (11.7)	–	1.00	–	1.00
>1	30 (25.2)	0.01	2.54 (1.25 ~ 5.18)	<0.01	2.76 (1.33 ~ 5.74)
**CAI with male sexual partners in the last 6 months**					
No	32 (18.4)	–	1.00	–	–
Yes	11 (19.6)	0.83	1.09 (0.51 ~ 2.33)	–	–
**Number of male casual partners in the last 6 months**					
≤2	30 (15.7)	–	1.00	–	1.00
>2	13 (33.3)	0.01	2.68 (1.24 ~ 5.80)	<0.01	3.02 (1.35 ~ 6.78)
**CAI with male casual partners in the last 6 months**					
No	39 (18.8)	–	1.00	–	–
Yes	4 (17.4)	0.87	0.91 (0.29 ~ 2.82)	–	–
**Number of male regular partners in the last 6 months**					
≤1	29 (15.4)	–	1.00	–	1.00
>1	14 (33.3)	0.01	2.74 (1.29 ~ 5.83)	<0.01	3.37 (1.51 ~ 7.51)
**CAI with male regular partners in the last 6 months**					
No	33 (18.2)	–	1.00	–	–
Yes	10 (20.4)	0.73	1.15 (0.52 ~ 2.54)	–	–
**Experienced sexual violence in the last 6 months**					
No or unsure	40 (17.9)	–	1.00	–	–
Yes	3 (50.0)	0.07	4.60 (0.90 ~ 23.63)	–	–
**Perpetrated sexual violence against others in the last 6 months**					
No or unsure	41 (18.1)	–	1.00	–	–
Yes	2 (66.7)	0.08	9.07 (0.80 ~ 102.46)	–	–
**PrEP used in the last 6 months**					
Yes	2 (18.2)	–	1.00	–	–
No	41 (18.7)	0.96	1.04 (0.22 ~ 4.98)	–	–
**PEP used in the last 6 months**					
Yes	3 (37.5)	–	1.00	–	–
No	40 (18.0)	0.18	0.37 (0.08 ~ 1.60)	–	–

* Controlled for the effects of age, marital status, occupation, and personal monthly income.

The results of the multivariate logistic regression analysis, which adjusted for demographic factors, demonstrated that in comparison to those who did not seek sexual partners within the preceding 6 months, seeking sexual partners through online platforms (aOR = 4.85, 95%CI: 1.42–16.59) and offline venues (aOR = 5.28, 95% CI: 1.01–27.79) both appeared to positively influence the initial adoption of HIVST. Importantly, among MSM, those who predominantly sought sexual partners offline exhibited a heightened likelihood of initiating HIVST. Furthermore, individuals with more than one male sexual partners (aOR = 2.76, 95%CI: 1.33–5.74), more than two male casual partners (aOR = 3.02, 95%CI: 1.35–6.78), or more than one male regular partner (aOR = 3.37, 95%CI: 1.51–7.51) demonstrated an elevated probability of initiating HIVST, underscoring a moderately stronger inclination toward HIVST usage among those with a relatively higher number of sexual partners. Refer to Table 2 for details.

We performed an additional stratification of the variable “primary method of seeking sexual partners in the last 6 months” into two categories (online platforms, offline venues) to assess differences in the initial adoption of HIVST. However, the univariate logistic regression analysis revealed no statistically significant difference between those who sought sexual partners offline and those who used online platforms (OR = 1.06, 95% CI: 0.33–3.45).

## 4 Discussion

Since 2014, global efforts, including those in China, have been dedicated to the widespread promotion and exploration of HIVST services among key populations like MSM (2, 9, 10, 17). Previous research has highlighted the primary challenges in promoting HIVST among China’s MSM population as awareness, accessibility, and financial factors (30–32). However, the past decade has witnessed a significant transformation in the availability of HIVST services. Both public health and commercial sectors now offer a diverse range of HIVST services through online and offline channels, including local CDCs, CBOs, pharmacies, and e-commerce platforms. This expansion has substantially increased awareness and accessibility of HIVST within the Chinese MSM community. As a result, this group can now conveniently access a range of HIVST services that suit their preferences and financial means (11, 14, 18). Despite these advancements, there is a research gap regarding the factors, including barriers and facilitators, that could further enhance HIVST promotion within the context of comprehensive service coverage.

It is crucial to highlight that the follow-up periods for assessing outcomes in both the case and control groups were aligned with the operational phase of VCT services in Guangzhou, avoiding overlap with the service disruptions caused by the COVID-19 pandemic in early 2020. Consequently, participants in both groups were able to freely access HIVST and VCT services. The adoption of HIVST was not exclusively driven by the unavailability of VCT services during the pandemic. Among the prospective cohort of MSM, from which our study sample was derived, only 10.9% (230/2,115) of participants reported having no prior experience with HIVST in the baseline survey. While prior studies have highlighted the role of HIVST awareness in shaping utilization behaviors (33), the 230 MSM included in our study, all of whom with no prior HIVST experience, had already established awareness regarding the availability and access to HIVST services during the baseline survey. This observation further supports the assumption that HIVST service accessibility among MSM in Guangzhou has reached a saturation point (11–13). Despite earlier studies suggesting that service accessibility influences utilization rates positively (23), our findings indicate that merely 18.7% of participants attempted initial HIVST during the follow-up period. This rate is significantly lower than the results of surveys conducted during the initial phase of HIVST promotion in various Chinese cities (15). This disparity suggests that while conventional promotional strategies can somewhat improve MSM awareness of HIVST, their effectiveness in continuously increasing utilization rates might be limited. Consequently, it is crucial to explore innovative and tailored HIVST service models that address the unique characteristics of specific subgroups (24–26).

This study utilized a nested case-control analysis and discovered that MSM exhibiting a higher number of male sexual partners tended to be more inclined toward initially embracing HIVST, aligning with conclusions drawn from earlier cross-sectional investigations and meta-analysis (34–37). Among MSM who recently disclosed having over two male casual partners, the probability of adopting HIVST initially was significantly higher. This heightened probability may arise from their involvement in frequent informal liaisons, fostering increased risk sensitivity and incentivizing them toward HIVST contemplation. Furthermore, this inclination might stem from an arrangement with male casual partners to undertake self-testing either before or after sexual interactions, confirming the HIV-negative status of both parties (38, 39). Moreover, MSM who recently reported having more than one male regular partners were more inclined to initially embrace HIVST. This increased likelihood among individuals having more than one male regular partners could be linked to the comparatively stable and lasting nature of relationships among them. MSM engaged in several male regular partnerships frequently opt to withhold information about other sexual partners from certain partners. To avoid potential conflicts within relationships, they might choose for private HIV testing methods, such as self-testing, to monitor their risk and infection status. These findings indicate the possibility of creating customized HIVST services designed specifically for MSM engaged in several male sexual partnerships. For example, product designs could center on situations that involve testing prior to engaging in sexual activities with male casual partners. Additionally, promotional and marketing strategies could be targeted at MSM engaged in a greater number of regular male-to-male sexual relationships to cater to their distinct testing needs and preferences.

The results of this study also demonstrate that MSM primarily seeking sexual partners offline experience have a significantly higher likelihood of initiating HIVST. MSM who seek sexual partners in places such as parks, public restrooms, clubs, and bathhouses, and engage in sexual behaviors within those venues, are more prone to neglect safety precautions due to factors like on-site conditions and peer influences. This could even lead to condomless group sexual activities (40–42). Furthermore, when they are active in offline settings and forming connections with sexual partners, MSM face challenges in reducing HIV infection risks through awareness of their infection status or pre-sexual behavior testing. Thus, compared to the Internet and social networking platforms, seeking sexual partners offline inherently exposes MSM to a higher risk of HIV infection (43, 44). Perhaps MSM who habitually seek sexual partners through offline venues might overlook risks in the heat of the moment during sexual encounters, but may subsequently express health-related concerns upon recalling their actions or encountering relevant health educational materials. Recognizing the potential risks of HIV infection associated with condomless behavior during that time could act as a motivation for embracing HIVST. These findings indicate a requirement for investigating appropriate HIVST service models in offline contexts. For example, deploying Internet of Things (IoT)-based HIVST services in offline venues frequented by MSM or providing HIVST with the support of key individuals like operators or volunteers in these venues. Furthermore, tailoring the HIVST application and registration process to suit the routines and educational levels of typical offline venue attendees is essential. This approach ensures a direct connection with potential HIVST users, proving more effective than generic online campaigns. By streamlining the process, we can reduce the learning curve and actively engage MSM, thereby enhancing their willingness to adopt these services. Beyond behavioral factors, structural barriers significantly impede the accessibility and utilization of HIVST (45). Despite the extensive HIVST services offered by both non-profit and commercial sectors, the predominant online marketing approach disenfranchises older adult or low-educated MSM who are unfamiliar with Internet operations. This highlights an urgent need to develop services specifically tailored to accommodate the needs of this population.

This study has some limitations. Firstly, our study design did not fully account for potential confounders like healthcare access and participants’ socioeconomic status, which might impact the results. Additionally, the participants were selected from a prospective cohort focusing on HIV infection, with recruitment occurring at VCT clinics. This recruitment strategy could introduce a selection bias, potentially limiting the representativeness of our sample to the broader MSM population. Specifically, our study predominantly includes young-to-middle-aged MSM and may not fully account for those lacking testing experience or practicing irregular testing behaviors. Furthermore, the nested case-control design may not conclusively establish causality among the variables.

In conclusion, directing efforts toward MSM with a higher number of sexual partners, especially those involved in regular partnerships and those seeking partners offline, through the creation of personalized, adaptable, and inventive HIVST strategies, may enhance the adoption of HIVST among MSM. This strategy may offer advantages, particularly considering the existing high awareness and extensive service availability of HIVST for this demographic.

## Data Availability

The datasets presented in this article are not readily available because our institution has a data access and sharing policy that outlines the procedures for data storage, access, and eventual sharing or archiving of data. We are bound by these policies. Requests to access the datasets should be directed to Yuzhou Gu, yuchoukoo@foxmail.com.
